# Multimodal phenotypic classification of generalized anxiety and panic using structural MRI data and psychosocial factors: machine learning results from the German National Cohort (NAKO) study

**DOI:** 10.1038/s41398-026-04131-1

**Published:** 2026-05-28

**Authors:** Julian Gutzeit, Martin Weiß, Tierney Kuhn, Johanna Klinger-König, Fabian Streit, Christiane Jockwitz, Berit Brandes, Marvin N. Wright, Christoph M. Friedrich, Margarethe Woeckel, Rafael Mikolajczyk, Thomas Keil, Stefanie Castell, Philine Betker, Christopher L. Schlett, Till W. Bärnighausen, Fabian Bamberg, Matthias Günther, Jochen G. Hirsch, Tobias Pischon, Thoralf Niendorf, Michael F. Leitzmann, Patricia Bohmann, Kerstin Wirkner, Lilian Krist, Yanding Wang, Klaus Berger, Sebastian Walther, Hans J. Grabe, Jürgen Deckert, Svenja Caspers, Grit Hein, Angelika Erhardt-Lehmann

**Affiliations:** 1 Department of Psychiatry, Psychosomatic and Psychotherapy, Center of Mental Health, University Hospital Würzburg, Würzburg, Germany; 2 Department of Psychology III, University of Würzburg, Würzburg, Germany; 3 Department of Psychology I, University of Würzburg, Würzburg, Germany; 4 Department of Psychiatry and Psychotherapy, University Medicine Greifswald, Greifswald, Germany; 5 Department of Genetic Epidemiology in Psychiatry, Central Institute of Mental Health, University of Heidelberg, Medical Faculty Mannheim, Mannheim, Germany; 6 Department of Psychiatry and Psychotherapy, Central Institute of Mental Health, Medical Faculty Mannheim, University of Heidelberg, Mannheim, Germany; 7 Hector Institute for Artificial Intelligence in Psychiatry, Central Institute of Mental Health, Medical Faculty Mannheim, University of Heidelberg, Mannheim, Germany; 8 German Center for Mental Health (DZPG), Partner Site Mannheim, Heidelberg - Ulm, Germany; 9 Institute for Anatomy I, Medical Faculty & Hospital Düsseldorf, Heinrich-Heine-University, Düsseldorf, Germany; 10 Institute of Neuroscience and Medicine (INM-1), Research Centre Jülich, Jülich, Germany; 11 Leibniz Institute for Prevention Research and Epidemiology—BIPS, Bremen, Germany; 12 Faculty of Mathematics and Computer Science, University of Bremen, Bremen, Germany; 13 Department of Public Health, University of Copenhagen, Copenhagen, Denmark; 14 University Hospital Essen, Institute for Medical Informatics, Biometry and Epidemiology (IMIBE), Essen, Germany; 15 University of Applied Sciences and Arts Dortmund (FH Dortmund), Department of Computer Science, Dortmund, Germany; 16 Institute of Epidemiology, Helmholtz Zentrum München, German Research Center for Environmental Health (GmbH), Neuherberg, Germany; 17 Department of Psychiatry and Psychotherapy, LMU University Hospital, LMU Munich, Munich, Germany; 18 Institute of Medical Epidemiology, Biometrics and Informatics, Medical Faculty of the Martin-Luther University Halle-Wittenberg, Halle, Wittenberg, Germany; 19 German Center for Mental Health (DZPG), Site Halle-Jena-Magdeburg, Halle (Saale), Germany; 20 Center for Intervention and Research on adaptive and maladaptive brain - Circuits underlying mental health (C-I-R-C), Halle-Jena-Magdeburg, Halle (Saale), Germany; 21 Institute of Social Medicine, Epidemiology and Health Economics, Charité-Universitätsmedizin Berlin, Berlin, Germany; 22 Institute of Clinical Epidemiology and Biometry, University of Würzburg, Würzburg, Germany; 23 State Institute of Health I, Bavarian Health and Food Safety Authority, Erlangen, Germany; 24 Department for Epidemiology, Helmholtz Centre for Infection Research (HZI), Brunswick, Germany; 25 Department of Diagnostic and Interventional Radiology, Medical Center–University of Freiburg, Faculty of Medicine, University of Freiburg, Freiburg, Germany; 26 Heidelberg Institute of Global Health (HIGH), Medical Faculty and University Hospital, Heidelberg University, Heidelberg, Germany; 27 Department of Global Health and Population, Harvard T.H. Chan School of Public Health, Harvard University, Boston, USA; 28 Africa Health Research Institute (AHRI), Somkhele and Durban, Durban, South Africa; 29 Fraunhofer Institute for Digital Medicine MEVIS, Bremen, Germany; 30 Max-Delbrück-Center for Molecular Medicine in the Helmholtz Association (MDC), Molecular Epidemiology Research Group, Berlin, Germany; 31 Max-Delbrück-Center for Molecular Medicine in the Helmholtz Association (MDC) Biobank Technology Platform, Berlin, Germany; 32 Charité – Universitätsmedizin Berlin, corporate member of Freie Universität Berlin and Humboldt-Universität zu Berlin, Berlin, Germany; 33 Berlin Ultrahigh Field Facility (B.U.F.F.), Max Delbrück Center for Molecular Medicine in the Helmholtz Association, Berlin, Germany; 34 Institute for Epidemiology and Preventive Medicine, University of Regensburg, Regensburg, Germany; 35 Department of Neurology, medbo District Hospital and University Hospital of Regensburg, Regensburg, Germany; 36 Leipzig Research Centre for Civilization Diseases, Leipzig University, Leipzig, Germany; 37 Institute for Medical Information Processing, Biometry, and Epidemiology (IBE), Faculty of Medicine, LMU Munich, Pettenkofer School of Public Health, Munich, Germany; 38 Institute of Epidemiology and Social Medicine, University of Münster, Münster, Germany; 39 German Centre for Neurodegenerative Diseases (DZNE), Site Rostock/Greifswald, Greifswald, Germany; 40 Max Planck Institute of Psychiatry, Munich, Germany

**Keywords:** Diagnostic markers, Neuroscience, Psychology

## Abstract

Anxiety disorders are common and impairing mental health conditions. Using data from 26,378 adults in the German National Cohort Study (NAKO), we investigated psychosocial and neuroimaging predictors of generalized anxiety disorder (GAD) symptoms and panic attacks. We conducted machine-learning analyses of 246 regions of interest from whole-brain imaging data in combination with psychosocial variables. Neuroimaging data alone showed suboptimal classification performance, whereas psychosocial variables alone - particularly depressive symptoms, stress, and childhood trauma - achieved the strongest discrimination for GAD symptoms and panic attacks. Adding neuroimaging features to psychosocial models modestly improved unbalanced accuracy and specificity by reducing false-positive classifications, indicating a conditional and complementary contribution of neuroanatomical information. Within the multivariate models, features from anxiety-related circuits, including the amygdala and superior parietal lobule, were consistently selected. Overall, these findings suggest that psychosocial factors dominate classification of anxiety outcomes, while structural MRI measures may provide complementary information within multimodal frameworks aimed at refining classification and supporting the development of individualized risk profiles to guide tailored therapeutic and preventive strategies.

## Introduction

Anxiety disorders are common and seriously impairing disorders, with an estimated lifetime prevalence up to 20% [1, 2]. A substantial proportion of anxiety disorders remain undetected or misrecognized in routine healthcare, largely due to symptom overlap with other mental disorders and with somatic diseases, particularly in primary care settings [3, 4]. This highlights the potential value of objective biomarkers that could complement clinical evaluations, improve diagnostic precision, and facilitate earlier identification of biologically distinct patient subgroups, thereby informing more targeted treatment strategies [5–7].

Generalized anxiety disorder (GAD) occurs with an estimated prevalence of 4–6% and is characterized by excessive, persistent and uncontrollable anxiety and worrying that is associated with nervousness, feelings of threatening uncertainty, and somatic complaints like muscular tensions and physiological hyperarousal [2, 8]. Beyond its high prevalence, GAD is considered one of the most impairing anxiety disorders, as it is associated with marked functional limitations, reduced quality of life, and elevated healthcare utilization [9, 10]. Importantly, GAD rarely occurs in isolation: it is highly comorbid with major depressive disorder, other anxiety disorders, and various somatic conditions [1]. This comorbidity reflects shared genetic and environmental vulnerabilities as well as overlapping neurobiological mechanisms [11], positioning GAD as a central disorder within the internalizing spectrum. Because of its chronic course, frequent treatment resistance, and role as a risk factor for the onset or persistence of depression and related conditions, GAD represents a pivotal target for research into reliable biomarkers that may improve diagnostic specificity and inform more effective, personalized treatment approaches.

Panic attacks represent a core symptom of panic disorder according to diagnostic criteria; however, they occur frequently in all anxiety disorders, and are strongly linked to general psychopathology as a separate dimension across mental conditions [12]. Panic attacks are defined as sudden and brief episodes of extreme anxiety as well as somatic stress symptoms, which in the case of mental disorders are inappropriate or unrealistically exaggerated compared to the target situation. Affected individuals tend to develop a fear of reexperiencing a panic attack that is associated with avoidance behavior and further negative behavioral changes, high distress and consequently individual burden [3, 13]. Prospective data suggest that panic attacks constitute a risk factor not just for the future development of any anxiety disorder, but also mood and substance use disorders [14]. Individuals experiencing panic attacks are at greater risk for increased persistence of mental disorders and impaired functioning, which underscores the importance of preventive treatment and early diagnosis of panic attacks to improve long-term outcomes [15]. Within the complex phenotypic composition of anxiety disorders, panic attacks represent characteristic and well-defined symptoms that can be interrogated using validated scales and attributed to biological anxiety circuits [16].

Both GAD and panic attacks are highly comorbid with other psychiatric conditions, most notably with depression [1]. This high comorbidity can be partly explained by shared neurobiological mechanisms, e.g., by overlapping environmental and genetic risk factors, as demonstrated in recent cross-disorder genome-wide association studies for pathological anxiety and depression [17, 18]. In addition, childhood adversity is one of the environmental factors potentially influencing brain morphology and significantly increasing the prevalence of anxiety disorders and depression [19, 20]. Therefore, it is crucial to incorporate these factors into our models to capture the complexity of anxiety-related phenotypes and improve the accuracy of classification analyses.

Several previous studies have investigated neuroimaging data as a promising candidate for biomarker identification. Lower gray matter volumes in bilateral orbitofrontal cortex and ventrolateral prefrontal cortex have been found to correlate with the General Distress dimension of the Tri-level Model (representing transdiagnostic depression and anxiety symptoms) [21]. Furthermore, higher gray matter volume in the amygdala has repeatedly been associated with GAD [22–24]. Functional imaging studies also consistently provide evidence that patients suffering from anxiety disorders display increased reactivity in the amygdala in response to negative emotional stimuli, along with insufficient prefrontal control [25]. Unfortunately, none of the biomarkers identified to date has demonstrated a sufficiently reliable predictive value to be used clinically [5, 25].

This may be partly due to limitations in previous research, including small sample sizes, heterogeneous analytical and clinical approaches, and relatively simple statistical methods that may not detect subtle associations in the large, high-dimensional data produced by neuroimaging. Machine learning algorithms, however, are particularly effective in analyzing high-dimensional data [26, 27], such as neuroimaging data, to identify complex patterns associated with conditions like GAD and panic attacks. Classifiers trained on the gray matter volume of anxiety-associated regions of interest (ROIs) in adolescent subjects achieved moderate predictive^1^ value for anxiety-disorder diagnosis in early adulthood [6]. A similar approach achieved robust (albeit modest) performance when classifying panic disorders vs. healthy controls based on subcortical volumes and cortical thickness and surface area [28].

Previous MRI-based machine learning studies of adult and adolescent anxiety have only used moderately large samples with imaging data from several hundred individuals [5, 6, 28]. Increasing the sample size by an order of magnitude can substantially increase the robustness of the findings by reducing the risk of overfitting [27], which may have contributed to the failure of certain models to replicate their predictive validity when evaluated on different data sets not used during training [5]. Therefore, the current paper builds on previous research by applying machine learning techniques to classify GAD symptoms and panic attacks, in a very large dataset that includes neuroimaging data from 26,378 adults taken from the German National Cohort Study (NAKO). To provide context for the subsequent machine learning analyses, we first conducted conventional correlational analyses on theory-driven, preselected neuroimaging variables while accounting for known confounders. This step served to illustrate the limitations of traditional linear approaches in detecting meaningful brain–behavior associations, thereby highlighting the added value of multivariate machine learning methods for capturing complex patterns in the data. Building on this conventional baseline approach, we subsequently performed multiple machine learning analyses (support vector machines with either a radial or linear kernel, random forests, elastic net regression), using the full set of whole-brain gray matter volume data without any regional preselection. This approach enables a more comprehensive, unbiased exploration of the brain, allowing the model to identify potential patterns that might be overlooked in region-specific analyses, and is consistent with the nomothetic networks psychiatry framework, which advocates integrating brain-based and psychosocial information into data-driven models of psychiatric classification [29].

## Methods

### Dataset and study population

Demographic, psychometric and brain-imaging data were taken from NAKO. Baseline assessment was conducted between 2014 and 2019, with the goal of investigating risk factors for a wide range of physical and mental chronic conditions, including depression, stress and anxiety symptoms. NAKO collected biomedical and questionnaire data from 205,415 persons living in Germany aged 19–74 who were chosen at random from compulsory registries of residents in 16 regions across Germany. This study analyzed the subsample of 30,927 participants who also later completed whole-body 3 T magnetic resonance imaging [30]. All participants gave written informed consent, and the data transfer was approved by the Use and Access Committee of NAKO. All NAKO study documents have been approved by all responsible local ethical committees and are revised regularly and adapted as needed. The sample size was not determined by an a priori power calculation, as this was a secondary analysis of an existing population-based cohort.

The two outcomes of interest in this study were clinically meaningful GAD symptoms and lifetime panic attacks. GAD symptoms were assessed using the established GAD-7 scale, which consists of seven items (yielding a maximum sum score of 21) to measure symptom load over the past four weeks. This measure has high reliability (Cronbach’s α = 0.83–0.93 in heterogeneous psychiatric samples) and demonstrates good convergent and discriminant validity [31]. We used the widely accepted GAD-7 ≥ 10 cutoff to define clinically meaningful GAD symptoms, as this threshold has been validated in large epidemiological and clinical studies, showing high sensitivity (~ 89%) and specificity (~ 82%) for detecting generalized anxiety disorder [32]. Employing this categorical definition allowed us to model the classification of symptom levels that are clinically relevant, rather than subthreshold anxiety that may be less stable and less informative for clinical decision-making. This approach also enhances comparability with previous research, where the same cutoff has been consistently used to identify probable GAD cases. Lifetime panic attacks were defined as having experienced one or more panic attacks in the past four weeks in addition to at least one previous lifetime panic attack. The PHQ-Panic scale shows good sensitivity (91%) and specificity (88%) and good convergent validity [33]. Because only the first part of the PHQ-Panic scale was administered, we could determine the presence of current and prior panic attacks, the mode of occurrence, and associated disability, but not a full diagnosis of panic disorder.

The demographic and psychometric predictors in our study included eight psychosocial variables in total: age, sex, number of lifetime cigarettes smoked, symptoms of GAD (GAD-7 score; only for classifying panic attacks), panic attacks (PHQ-Panic; only for classifying GAD symptoms), depression (current PHQ-9 score [34]), stress (current PHQ-Stress score [35]), and childhood trauma (childhood trauma screener, CT-S [36]). All psychosocial variables were collected by self-administered touchscreen questionnaires during an in-person baseline examination in the study centers [30]. The detailed description of psychometric and emotional scales within the NAKO is available elsewhere (GAD-7, panic and stress [37], childhood trauma [38], depression [39]). After excluding participants with missing values for any of the predictors or outcomes, 26,378 participants were included in the study.

### MRI acquisition and preprocessing

Structural MRI data for all participants was obtained using 3 T scanners (Magnetom Skyra; Siemens Healthcare, Erlangen, Germany) across five NAKO study centers (Essen, Neubrandenburg, Berlin, Augsburg, and Heidelberg/Mannheim). Images were acquired using a T1-weighted 3D MPRAGE sequence (1.0 × 1.0 × 1.0 mm (isotropic) voxel; sagittal orientation; repetition time msec/echo time msec/inversion time msec, 2300/2.98/900; 9° flip angle [40]). The T1 images were segmented, normalized, and smoothed.

An automated quality-control pipeline was used to assess each image’s sharpness, global and local signal-to-noise ratio, maximum and average estimates for structured image noise and Nyquist ghosting levels, and geometric ratio between foreground and background. Subsequently, board-certified radiologists performed a visual rating using a three-point Likert scale that considered anatomical coverage, minimum differentiable structures, and the presence of artifacts, and excluded any images rated as ‘Poor’ [41].

The gray matter volumes of the 246 brain areas defined by the Julich-Brain Cytoarchitectonic Atlas (https://atlases.ebrains.eu/ [42] using the software Computational Anatomy Toolbox (CAT12v8; https://neuro-jena.github.io/cat/) were extracted from those T1-weighted images that passed quality control. In total, 246 neuroimaging variables (ROIs) were derived, encompassing cortical and subcortical volumes (including the limbic system), surface areas, and mean cortical thickness values. These were included in the neuroimaging and combined variable sets described below.

### Machine-learning classification

The classification analysis was conducted in R, version 4.4.3 [43] using four models from the machine learning package *caret*, version 6.0–94 [44]: support vector machines with either a radial (SVM-R) or linear kernel (SVM-L), random forests (RF), and elastic net regression (ELNET). Each model was trained in two distinct analyses to make binary predictions regarding: (a) the presence versus absence of clinically relevant GAD symptoms (defined as GAD-7 ≥ 10, labelled as GAD symptoms), and (b) the presence versus absence of combined current and lifetime panic attacks (according to the first two items of the PHQ-Panic scale)^2^. Analyses were conducted using three variable sets: (1) 246 neuroimaging variables (N), (2) 8 psychosocial variables (P) [sex, age, number of lifetime cigarettes, depression, stress, childhood trauma, and either panic attacks (for classifying GAD symptoms) or GAD-7 score (for classifying panic attacks)], and (3) a combined set of 254 variables (P + N). This explicit separation was chosen for comparability with previous work [6]. All variables were standardized to *z*-scores.

The participants were randomly divided into a training set with *n* = 21,102 subjects and a test set with *n* = 5,276 subjects. A linear confound regression was used to regress out the impact of sex, age, total intracranial volume, lifetime cigarettes smoked, childhood trauma, and scanner site on gray matter volume in the training dataset [45]. Scanner site was included as a categorical factor (dummy coded), thereby accounting for potential site effects in the residualized data. We computed multiple linear regressions with these potential confounding variables for each brain area volume. Only the residuals of these regression analyses were included in the machine-learning models. To prevent data leakage – that is, the unintended use of test data information inflating model performance – the regression weights estimated from the training dataset were also applied to control for confounds in the test dataset [46].

Hyperparameter tuning was performed using 5-fold cross-validation, with the area under the receiver operating characteristic curve (AUROC) as the outcome measure to maximize. For the SVM-L, the regularization constant was varied across nine values on a base-2 logarithmic scale, ranging from 2⁻⁸ (0.0039) up to 2⁸ (256). For the SVM-R, the kernel width parameter sigma was not tuned manually but estimated automatically from the data using the sigest procedure from the kernlab package [47], which derives a plausible value based on the distribution of pairwise distances between observations. With sigma fixed in this way, the regularization constant was tuned across ten exponentially spaced values on the log-2 scale, ranging from 0.25 up to 128. For the RF models implemented with the *ranger* engine [48], *caret*’s automatic tuning procedure with a tuning length of ten was applied. This created a tuning grid where the number of candidate predictors tried at each split (mtry) spanned 10 values between 1 and the total number of predictors. For each of these values, both the “gini” and “extratrees” splitting rules were considered, while the minimum node size was kept constant at 1. For elastic net regression, *caret* tuned the mixing parameter α over ten values between ridge (α = 0) and lasso (α = 1). For each α, the package *glmnet* [49] automatically generated a logarithmically spaced sequence of 100 λ values, ranging from λmax (the value that shrinks all coefficients to zero) down to λmin, set at 0.0001 × λmax (for n > p). Across all algorithms, the best-performing hyperparameter configuration was identified based on cross-validated AUROC, and the final model was then refitted on the full training dataset before evaluation on the independent test set. The best-fitting tuning parameters are reported in Extended Data Table S8. Consistent with population epidemiology, only a minority of participants had GAD-7 score ≥ 10 or panic attacks (see Results section). Because of this, the data were substantially imbalanced, which can bias machine learning classifiers toward the majority class. To address this, we applied random undersampling of the control group within each fold of cross-validation [50]. Specifically, for every training fold, a random subsample of participants without the target condition was drawn so that class sizes were balanced. This procedure was repeated independently in each fold, ensuring that nearly all control participants contributed to model training across iterations. By reducing the dominance of the majority class in each fold, this strategy prevented skewed classification performance while still leveraging the full sample across resampling cycles (^for a similar approach, see 28^).

After training, the model with the best performance (when using optimal hyperparameters) was identified for each combination of classification target and variable set. We computed several performance measures on the confusion matrices of the best models for each data set (N, P, P + N). Additionally, we assessed variable importance in the winning P + N models by computing the AUROC for each predictor. This approach tests each predictor individually and computes the AUROC based on predictions made using only that predictor and the model. This method has the advantage of providing the predictive importance of each single predictor for each model and allows for comparisons across multiple classification models [51]. Higher values indicate stronger associations with the outcome and can be interpreted as indicating effect sizes. To further assess the direction of these associations, we computed simpler logistic regression models using the ten most important predictors. In contrast to separate Pearson correlations between individual predictors and various outcomes, these analyses test the effect of all ten predictors simultaneously.

For completeness, we additionally conducted conventional association-based analyses of a theory-driven, preselected set of neuroimaging variables, which are reported in the Supplementary Material. Using sex-stratified Pearson correlations, we examined univariate associations between selected global, regional, and network-level structural MRI measures and clinically relevant GAD symptoms and panic attacks, with adjustment for age, overlapping psychopathology, childhood trauma, and scanner site. Full methodological details and results are provided in the Supplementary Material S1.

## Results

Of all participants, 4.4% (*n* = 1,161) reported a GAD-7 score ≥ 10, indicating clinically meaningful anxiety symptoms. Additionally, 3.8% (*n* = 1,002) reported experiencing panic attacks. For an overview of all participant characteristics, see Table 1. GAD and panic attacks were moderately related (*r* = 0.31, *p* < 0.001).

**Table 1:** Demographic characteristics of analyzed study participants with valid data (*n* = 26,378).

Variable		GAD symptoms		Panic attacks		Total
		no	yes	no	yes	
Participants	n	25,217	1,161	25,376	1,002	26,378
Sex	Men	14,530 (57.6%)	502 (43.2%)	14,619 (57.6%)	413 (41.2%)	15,032 (57.0%)
	Women	10,687 (42.4%)	659 (56.8%)	10,757 (42.4%)	589 (58.8%)	11,346 (43.0%)
Age	mean (SD)	47.5 (12.3)	45.8 (11.8)	47.5 (12.2)	46.8 (12.4)	47.4 (12.2)
	18–29	2,855 (11.3%)	148 (12.7%)	2,877 (11.3%)	126 (12.6%)	3,003 (11.4%)
	30–39	3,163 (12.5%)	179 (15.4%)	3,211 (12.7%)	131 (13.1%)	3,342 (12.7%)
	40–49	7,672 (30.4%)	353 (30.4%)	7,728 (30.5%)	297 (29.6%)	8,025 (30.4%)
	50–59	6,864 (27.2%)	324 (27.9%)	6,910 (27.2%)	278 (27.7%)	7,188 (27.2%)
	60–74	4,663 (18.5%)	157 (13.5%)	4,650 (18.3%)	170 (17.0%)	4,820 (18.3%)
GAD-7	mean (SD)	2.6 (2.2)	12.7 (2.7)	2.8 (2.8)	8.6 (4.6)	3.0 (3.1)
GAD-7 ≥ 10	healthy	25,217 (100.0%)	0 (0%)	24,580 (96.9%)	637 (63.6%)	25,217 (95.6%)
	anxious	0 (0%)	1,161 (100.0%)	796 (3.1%)	365 (36.4%)	1,161 (4.4%)
Lifetime Panic Attacks	no	24,580 (97.5%)	796 (68.6%)	25376 (100.0%)	0 (0%)	25,376 (96.2%)
	yes	637 (2.5%)	365 (31.4%)	0 (0%)	1,002 (100.0%)	1,002 (3.8%)
Lifetime Cigarettes	mean (SD)	6.0 (192.2)	10.6 (260.8)	6.0 (191.6)	11.6 (280.7)	6.2 (195.7)
Depressive Symptoms (PHQ-9)	mean (SD)	3.3 (2.9)	12.8 (5.2)	3.5 (3.3)	9.4 (5.8)	3.7 (3.6)
Stress	mean (SD)	3.1 (2.7)	9.2 (3.6)	3.2 (2.8)	7.5 (4.1)	3.4 (3.0)
Childhood Trauma Sum Score (CT-S)	mean (SD)	7.0 (2.4)	8.8 (3.7)	7.1 (2.5)	8.5 (3.5)	7.1 (2.5)

**Fig. 1:**
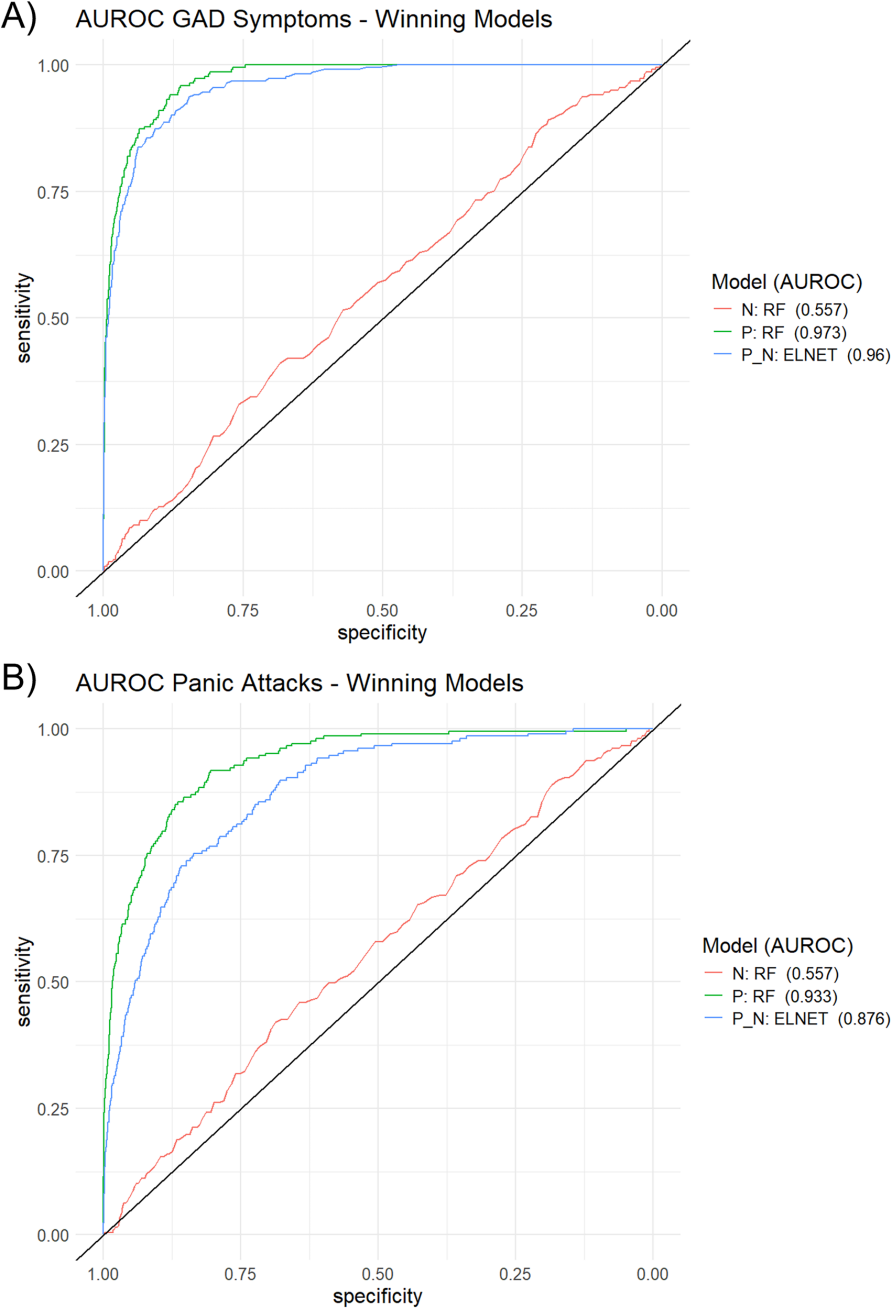
Model Classification Performance for GAD Symptoms and Panic Attacks. AUROC curves for each winning model are shown for the following datasets: 246 neuroimaging variables (N), consisting of whole-brain imaging variables from the Juelich Atlas [42]; eight psychosocial variables (P) – including sex, age, lifetime cigarettes smoked, depression, stress, childhood trauma, and either panic attacks (PA) for classifying clinically relevant GAD symptoms or the GAD-7 score for classifying PA; and the combination of both datasets (P + N; total of 254 variables). Panel **A** displays results for GAD symptoms, and Panel **B** for panic attacks. RF: random forest, ELNET: elastic net regression.

We used machine learning to identify the brain structures and psychosocial variables that are most important for predicting these clinically relevant outcomes. As can be seen in Table 2, the random forest classifier outperformed other P-models (i.e., models using only psychosocial variables as input data) for both GAD symptoms (AUROC = 0.973) and panic attacks (AUROC = 0.933) in the test dataset. The random forest classifier performed best on the datasets containing only neuroimaging data (N-models) but still showed relatively poor performances for classifying GAD symptoms (AUROC = 0.557) and panic attacks (AUROC = 0.557). ELNET showed the best performance using the combination of neuroimaging data and psychosocial variables (P + N) to classify GAD symptoms (AUROC = 0.959) and panic attacks (AUROC = 0.876), but did not outperform random forest P-models. AUROCs for the winning models for each dataset (P, N, P + N) are depicted in Fig. 1.

**Table 2:** Area under receiver operating characteristic curve (AUROC) values for each model and each algorithm.

	GAD Symptoms				Panic Attacks			
model	SVM-L	SVM-R	RF	ELNET	SVM-L	SVM-R	RF	ELNET
N	0.517	0.553	*0.557*	0.528	0.546	0.520	*0.557*	0.522
P	0.962	0.950	**0.973**	0.962	0.875	0.866	**0.933**	0.877
P + N	0.949	0.954	0.957	*0.959*	0.852	0.831	0.874	*0.876*

*N neuroimaging variables, P psychosocial and psychometric variables, SVM-L linear support vector machine, SVM-R radial support vector machine, RF random forest, ELNET elastic net regression*.

*The algorithms resulting in the highest AUROC for each model are printed in italic type and the highest value for each outcome in bold type*.

**Fig. 2:**
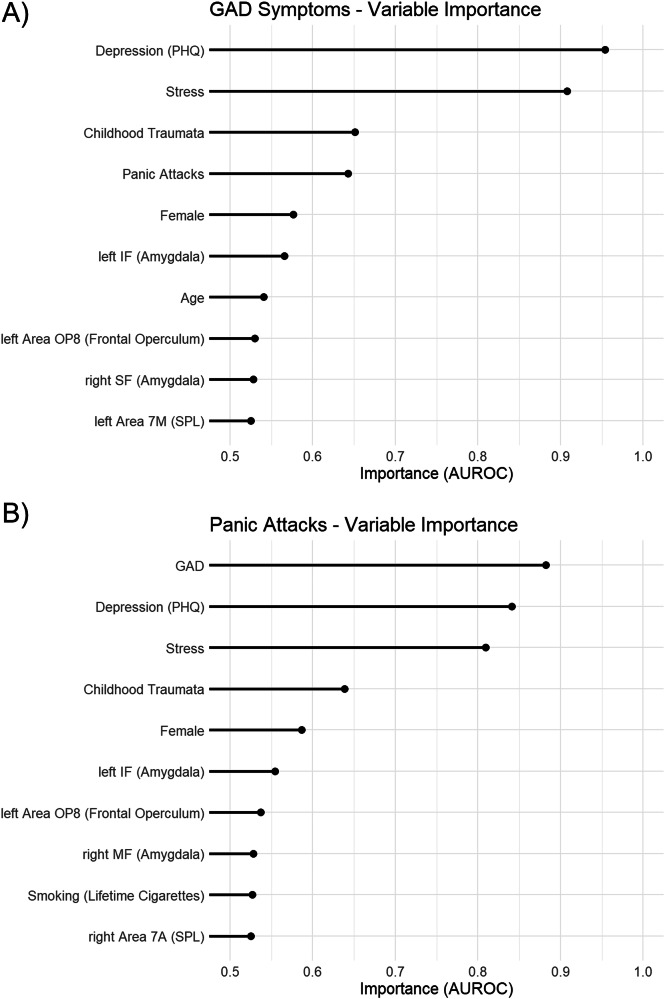
Top Variables for Classification of GAD Symptoms and Panic Attacks by Elastic Net Regression. Top 10 important variables (4%) for the elastic net classification of GAD (**A**) and panic attacks (**B**) symptoms. SPL: superior parietal lobule; IF: internal intermediate fiber masses of amygdala; MF: internal medial fiber masses of amygdala; SF: superficial amygdala; OP8: frontal operculum 8.

To investigate which variables had the highest predictive importance for the outcome variables, we computed the AUROC importance for the P + N models [51]. Variables with higher AUROC importance scores are considered more predictive. We depicted the top 10 variables (4% of all variables; for similar approach, see [52]) separately for GAD (Fig. 2A) and panic attacks (Fig. 2B). The importance scores for all variables of all winning models are provided in the Extended Data as follows: the winning P models are detailed in S2 (GAD) and S3 (panic attacks); the winning N models in S4 (GAD) and S5 (panic attacks); and the winning P + N models in S6 (GAD) and S7 (panic attacks).

In addition to AUROC, we computed performance metrics derived from the confusion matrices for the top-performing models (P, N, P + N), as summarized in Table 3. Interestingly, while the elastic net P + N models did not achieve higher AUROCs compared to the random forest P models, both P + N models for GAD and panic attacks exhibited higher unbalanced accuracy and positive predictive value (i.e., the likelihood that a positive classification is a true positive, also referred to as precision) as well as higher specificity values compared to random forest P models.

**Table 3:** Confusion matrices for the models with the highest AUROC.

	GAD Symptoms			Panic Attacks		
Metric	N (RF)	P (RF)	P + N (ELNET)	N (RF)	P(RF)	P + N (ELNET)
Accuracy	0.499	0.896	0.905	0.544	0.802	0.846
Kappa	0.011	0.383	0.398	0.011	0.214	0.221
AccuracyLower	0.485	0.887	0.897	0.530	0.791	0.836
AccuracyUpper	0.512	0.904	0.913	0.557	0.813	0.856
AccuracyNull	0.958	0.958	0.958	0.961	0.961	0.961
AccuracyPValue	1.000	1.000	1.000	1.000	1.000	1.000
McnemarPValue	0.000	0.000	0.000	0.000	0.000	0.000
Sensitivity	0.575	0.910	0.873	0.522	0.918	0.729
Specificity	0.496	0.895	0.907	0.544	0.797	0.851
Pos Pred Value	0.047	0.275	0.290	0.045	0.156	0.166
Neg Pred Value	0.964	0.996	0.994	0.965	0.996	0.987
Precision	0.047	0.275	0.290	0.045	0.156	0.166
Recall	0.575	0.910	0.873	0.522	0.918	0.729
F1	0.088	0.422	0.436	0.082	0.267	0.271
Prevalence	0.042	0.042	0.042	0.039	0.039	0.039
Detection Rate	0.024	0.038	0.037	0.020	0.036	0.029
Detection Prevalence	0.507	0.139	0.126	0.458	0.231	0.172
Balanced Accuracy	0.535	0.902	0.890	0.533	0.858	0.790
AUROC	0.557	0.973	0.959	0.557	0.933	0.876

*RF random forest, ELNET elastic net regression*.

The most important psychosocial variables (P) for classifying individuals with clinically relevant symptoms of GAD were depressive symptoms (PHQ-9 score), psychological distress (dimensional PHQ-Stress), childhood trauma (dimensional CT-S), panic attacks, female sex, and age (Fig. 2A). The most important neuroimaging (N) variables were gray matter volume in the regions of the left internal intermediate fiber masses (IF) of the amygdala, the left area OP8 in the frontal operculum, the right superficial fiber masses (SF) of the amygdala, and the left area 7 M in the superior parietal lobule.

For the classification of panic attacks, the most important psychosocial variables (P) were generalized anxiety disorder symptoms (GAD-7 score), depressive symptoms (PHQ-9 score), psychological distress (dimensional PHQ-Stress), childhood trauma (dimensional CT-S), female sex, and lifetime cigarettes (Fig. 2B). The most important neuroimaging (N) variables were gray matter volume in the regions of the left internal intermediate fiber masses of the amygdala, the left area OP8 in the frontal operculum, the right internal medial fiber masses of the amygdala, and the right area 7 A in the superior parietal lobule.

To evaluate the direction of these associations, the results of the simple logistic regression models for outcomes and the predictor variables are depicted in Table 4.

**Table 4:** Coefficients and Odds-Ratios of the logistic regression on GAD symptoms and panic attacks with top 10 variables of the winning P + N model.

GAD symptoms					
Independent variable	Estimate	SE	z	*p*	OR [95%CI]
Depression (PHQ)	**1.34**	**0.04**	**33.44**	**<0.001**	**3.84 [3.55, 4.15]**
Stress	**0.64**	**0.04**	**16.03**	**<0.001**	**1.90 [1.75, 2.05]**
Childhood Trauma	0.01	0.03	0.24	0.813	1.01 [0.94, 1.08]
Panic Attacks	**1.08**	**0.11**	**9.71**	**<0.001**	**2.93 [2.36, 3.64]**
Female	0.02	0.05	0.40	0.686	1.02 [0.93, 1.12]
Left IF (Amygdala)	0.07	0.04	1.75	0.080	1.08 [0.99, 1.17]
Age	−0.06	0.05	−1.25	0.212	0.94 [0.86, 1.04]
Left Area OP8 (Frontal Operculum)	−0.07	0.05	−1.22	0.221	0.94 [0.84, 1.04]
Right SF (Amygdala)	−**0.11**	**0.05**	−**1.98**	**0.048**	**0.90 [0.81, 1.00]**
Left Area 7 M (SPL)	**0.16**	**0.05**	**3.05**	**0.002**	**1.18 [1.06, 1.31]**
**Panic Attacks**					
GAD	**0.76**	**0.04**	**18.87**	**<0.001**	**2.15 [1.98, 2.33]**
Depression (PHQ)	**0.13**	**0.04**	**3.25**	**0.001**	**1.14 [1.05, 1.23]**
Stress	**0.24**	**0.04**	**6.21**	**<0.001**	**1.27 [1.18, 1.36]**
Childhood Trauma	**0.12**	**0.03**	**4.01**	**<0.001**	**1.12 [1.06, 1.19]**
Female	**0.10**	**0.04**	**2.56**	**0.010**	**1.11 [1.03, 1.20]**
Left IF (Amygdala)	−0.02	0.04	−0.47	0.635	0.98 [0.91, 1.06]
Left Area OP8 (Frontal Operculum)	−0.08	0.04	−1.95	0.051	0.92 [0.85, 1.00]
Right MF (Amygdala)	**0.08**	**0.04**	**2.06**	**0.040**	**1.08 [1.00, 1.65]**
Smoking (Lifetime Cigarettes)	−0.02	0.03	−0.61	0.542	0.98 [0.92, 1.03]
Right Area 7 A (SPL)	−0.08	0.04	−1.77	0.076	0.93 [0.85, 1.01]

Significant predictors in bold. Gray matter volume data are confound regressed for sex, age, total intracranial volume, lifetime cigarettes smoked, childhood trauma, and scanner site.

SPL superior parietal lobule, SE standard error of the mean, OR odds ratio, CI confidence interval.

## Discussion

In the present study, we employed multiple machine learning models to model clinically relevant symptoms of GAD and panic attacks in a sample of 26,378 individuals. Although GAD and panic attacks were correlated in our sample, the association was only moderate (*r* = 0.31), indicating that the two outcomes are related but not redundant. To classify individuals with or without these symptoms, we utilized regional gray matter volume of a whole-brain parcellation, psychosocial variables (age, sex, number of lifetime cigarettes, symptoms of GAD, panic attacks, depression, stress, and childhood trauma), or a combination of both. We found that a random forest model using only psychosocial variables achieved the highest AUROC for classifying both individuals with GAD and panic attacks. ELNET models combining brain volume data and psychosocial variables demonstrated the highest unbalanced accuracy. Depressive symptoms, childhood trauma, psychological distress, and female sex were the psychosocial variables with the highest predictive importance for classifying clinically relevant GAD symptoms and panic attacks. Current and lifetime panic attacks were among the most important variables for classifying GAD symptoms, while GAD symptoms were the most important statistical predictor for classifying panic attacks. Although neuroanatomical features did not dominate overall predictive performance, several brain regions showed consistent importance within the multivariate models. Among brain regions, amygdala volume, areas in the frontal operculum, and the superior parietal lobule showed the highest importance for both symptom categories. We view the present results as a step toward a multimodal, nomothetic networks framework for psychiatric classification [29], demonstrating that combining psychosocial and neuroimaging features can improve discrimination of anxiety-related phenotypes, while leaving mechanistic pathway modeling to future work.

Generally, models using only psychosocial variables (such as other psychiatric symptoms, psychological stressors, and demographic variables) demonstrated the best-balanced performance in classifying clinically relevant GAD symptoms (Random Forest AUROC = 0.973) and panic attacks (Random Forest AUROC = 0.933). Gray matter volume data did not substantially improve predictive quality and even led to slightly worse performances (ELNET AUROC = 0.959 for GAD symptoms and 0.876 for panic attacks, respectively). These results align with a recent study by Chavanne et al. [6], who predicted the onset of anxiety disorders in adolescents using gray matter volume and psychometric data. In their study, psychometric variables alone had sufficient predictive power, and performance sometimes declined when brain volume data was included. Furthermore, our gray matter volume data alone showed only poor classification performance of GAD (Random Forest AUROC = 0.557) or panic attacks (Random Forest AUROC = 0.557), a finding in line with previous other machine learning studies [6, 28, 53, 54]. Importantly, although gray matter volume did not substantially improve overall discrimination, adding gray matter volume to psychosocial variables increased the unbalanced accuracy and specificity. This indicates that the ELNET model with a combination of gray matter volume and psychosocial variables might be more reliable in detecting negative cases, i.e. individuals without GAD or panic attacks, than models based on psychosocial variables alone. Thus, neuroanatomical features appear to contribute conditionally to model performance by refining classification boundaries rather than by driving overall classification accuracy. Accordingly, our findings align with those recently reported in the ENIGMA mega-analyses. In one study, Hilbert et al. [55] highlighted distinct cortical and subcortical structural changes in specific phobia, with pronounced differences based on subtype. Similarly, our results underscore the importance of subcortical volumes in anxiety-related psychopathology. Furthermore, Groenewold et al. [56] emphasize the role of subcortical structures, such as the putamen and pallidum, in anxiety disorders. In line with this work, the brain regions identified in our models likely reflect subtle, distributed neurobiological contributions that are detectable in multivariate frameworks, even when psychosocial variables dominate overall predictive performance.

In our case, variable importance analyses revealed that psychosocial characteristics generally held greater predictive power than variations in gray matter volume. Depressive symptoms were identified as the most important predictor for clinically relevant GAD symptoms and the second most important predictor for panic attacks. This finding aligns with existing research showing that anxiety disorders are frequently followed by depression [57, 58]. Moreover, there is substantial comorbidity between GAD and depression (with 45–98% of GAD patients suffering from both conditions [59]), and between panic disorder – defined as the disorder with panic attacks as a core symptom – and major depression (with about 50% of panic disorder patients suffering from a depressive episode [60]). Additionally, symptom overlap between GAD and depression as assessed with GAD-7 and PHQ-9 might contribute to relevant predictive power of depression for GAD symptoms. The most important predictors for classifying panic attacks were symptoms of GAD. Conversely, lifetime panic attacks ranked as the fourth most important variable for classifying symptoms of GAD. This relationship can, again, be explained by the high degree of comorbidity between these syndromes and shared neurobiological factors [59].

Interestingly, childhood trauma (i.e., experiences of abuse and neglect) emerged as a relatively important factor for classifying symptoms of both GAD and panic attacks. This aligns with previous research demonstrating that childhood trauma is a substantial risk factor for both syndromes [61–64]. It should be noted, however, that while the relatively complex ELNET classifier trained on the combination of psychosocial and neuroimaging data found associations with both GAD and panic attacks, the simple logistic regression models only demonstrated a significant association with panic attacks.

Additionally, female sex proved to be a relatively important variable for machine-learning model performance for both conditions. This finding is consistent with a substantial body of research showing that females are affected by anxiety and depressive disorders more frequently than males, and that machine-learning analyses can capture sex-related differences more flexibly [65–67]. Lower age showed relatively modest but notable importance for classifying GAD symptoms in the elastic net regression – although not statistically significant, the effect was descriptively in the same direction as in the logistic regressions. This finding could be explained by the typical age at onset of GAD, which is usually during middle/late adulthood [57, 68], and there is evidence that the intensity and number of symptoms might decrease with increasing age [69, 70]. Additionally, the lifetime number of cigarettes smoked showed a subtle relationship with panic attacks; however, this relationship was not significant in the simpler logistic regression. While studies suggest that smoking might be a risk factor for developing panic disorder and anxiety disorders in general [58, 71, 72], it is also possible that smoking develops as a coping mechanism for panic attacks [73]. In line with this, one recent systematic review on the prospective bidirectional association of smoking and anxiety pathology showed inconsistent results [74].

The amygdala displays a complex anatomical and cellular organization with several subregions having distinct functions and brain connections [75]. The individual internal fiber masses consist of medial (MF) and intermediate (IF) fiber bundles and separate the amygdaloid substructures [76]. The IF and MF are attached to the ventromedial part of the basomedial nucleus (BMA) of the laterobasal complex of the amygdala. The BMA has been shown to be the major target of ventral medial prefrontal cortex projections (mPFC), which constitute one top-down regulation pathway to control anxiety states [77]. Additionally, previous work implies the existence of a BMA microcircuit involved in fear acquisition, generalization, recall, and extinction in a distinct and sex-dependent manner [78]. In the present study, amygdala subregion volumes contributed consistently to multivariate model decisions, motivating a closer examination of their potential neurobiological relevance. Specifically, higher gray matter volume in the left amygdala IF was associated with clinically relevant GAD symptoms in the machine-learning models, while lower gray matter volume was linked to panic attacks. Although these associations did not emerge as strong standalone effects in conventional regression analyses, the opposing directions across outcomes may reflect distinct clinical profiles between GAD and panic attacks, with potentially greater involvement of mPFC–amygdala circuitry in GAD [79]. However, as the specific functional implications of the IF have not been evaluated so far, further studies are needed to clarify its utility as a phenotypic marker for different anxiety disorders.

The superficial amygdala (SF) is located close to the hippocampal formation. In the present study, lower gray matter volume of the SF contributed to the classification of clinically relevant GAD symptoms in the multivariate models. Studies investigating reactivity to emotional faces and activation of SF suggest its implication in social relevance processing and also fear-related memory [80, 81]. In general, previous research has yielded mixed findings regarding the relationship between amygdala gray matter volume and GAD. Some studies report increased amygdala volumes, while others show decreased volumes compared to healthy controls [24] (for a review see [82]). A recent study suggests that anomalies in amygdala volume among GAD patients may be driven by comorbid anxiety disorders, as individuals with GAD without comorbidities may not exhibit amygdala volume differences compared to healthy controls [83]. Thus, the relationship between amygdala gray matter volume anomalies and GAD may be complex and warrants further investigation in future research. Previous research has shown that smaller amygdala volume was associated with panic disorder [84] (for a review see [85]), which is in line with our findings of smaller gray matter volume in the left amygdala being an important classification variable for panic attacks. However, we also found *higher* gray matter volume of the right MF amygdala to be associated with panic attacks. These findings are in contrast to previous research and need further investigation.

Given they have different locations within the amygdala, the IF and MF might have individual implications in anxiety-related circuits, which need to be evaluated in further studies. So far, the evidence on volumetric associations with panic (and GAD) is mostly based on total amygdala gray matter volume measurements. In contrast, subnuclear and fiber-mass–specific gray matter volumes may capture more fine-grained neuroanatomical variation relevant for multivariate classification, for example functional adaptations in response to specific patterns of neural activity inside the amygdala subdivisions or in connection with other brain regions such as the mPFC. Using a detailed microstructural anatomical atlas defining relevant brain areas, like the Julich-Brain Atlas used in this study, could assist in further specifying the differential roles of specific substructures in GAD and panic attacks. In terms of laterality, sex might play a role, as a recent study reported clear sex-differences in the left and right amygdala with differential sex-specific genetic correlation only in the left amygdala [86].

We also found that reduced gray matter volume in the frontal operculum contributed to the classification of both GAD symptoms and panic attacks in the multivariate models, although these relationships were only found with the complex machine-learning analyses and were not significant in the simple logistic regressions. Area OP8 is located in the frontal part of the frontal operculum, the latter being part of a network controlling activity in other brain areas during performance of cognitive tasks in the sense of cognitive control [87]. Previous research has shown that the operculum is associated with negative cognition and worrying [88]. Excessive worry is the core symptom of GAD and worrying about future panic attacks is an important symptom of panic disorder [89]. Accordingly, variation in frontal operculum structure may reflect neural processes related to anxious rumination that are conditionally relevant within multivariate classification frameworks, rather than representing strong standalone markers of anxiety disorders. Whether such structural differences constitute predisposing factors or consequences of sustained worry cannot be determined from the present cross-sectional data. Last, we found that larger gray matter volume of the left area of the superior parietal lobule (SPL) was associated with GAD symptoms and smaller gray matter volume of the right area of the SPL was associated with panic attacks. Again, these relationships only emerged in the machine-learning model and were not significant in the logistic regression models, suggesting that SPL involvement may be subtle and context-dependent within multivariate patterns rather than detectable as isolated linear effects.

There has been some research showing that anxiety disorders might be associated with anomalies and activity changes in superior parietal regions [85, 90]. In summary, although the contribution of neuroimaging variables to the overall classification of GAD symptoms and panic attacks was limited, our analyses nonetheless detected brain areas implicated in anxiety-related circuits as conditionally informative features within multivariate models. This suggests that neuroanatomical structures may provide complementary information, rather than serving as strong standalone predictors, and could be considered as part of multimodal biomarker panels in future research. The somewhat lower performance of combined (P + N) models compared to psychosocial models alone can be explained by the fact that psychosocial variables capture a large share of the variance in anxiety risk, whereas structural imaging features are numerous but individually only weak predictors. When such high-dimensional, low-signal features are added to already strong predictors, algorithms like random forests may be prone to noise or overfitting, leading to small drops in accuracy. Penalized regression models, in contrast, integrated imaging and psychosocial features more effectively, yielding improvements in specificity and positive predictive value. Thus, while psychosocial measures demonstrated superior overall discrimination, neuroimaging features contributed selectively to particular aspects of classification performance and remain of interest for understanding the neurobiological architecture underlying anxiety phenotypes, rather than for immediate clinical classification.

As highlighted in recent reviews, brain-based predictors may capture latent endophenotypes and mechanistic markers not accessible through self-report, offering unique potential for early detection, individualized prognosis, and treatment stratification [26]. Even when their incremental predictive value is modest at the level of overall model performance, neuroimaging can complement behavioral assessments by providing biologically grounded insights into the heterogeneity of anxiety presentations and supporting precision psychiatry efforts. Furthermore, advances in single-subject prediction frameworks demonstrate that structural and functional MRI can, in principle, yield robust biomarkers of psychiatric risk and treatment response, though current limitations in sample size, generalizability, and methodological rigor still constrain immediate clinical translation [91]. Consistent with this perspective, our own results showed that the inclusion of neuroimaging variables alongside psychosocial measures improved model specificity. This suggests that while psychosocial data may capture the more dominant sources of variance in anxiety risk, neuroimaging features provide complementary, biologically grounded information that helps refine classification boundaries. In clinical terms, such specificity gains may prove valuable in differentiating subgroups of patients or in tailoring interventions, even if the overall predictive accuracy of imaging alone remains more modest.

The present findings have direct implications for how clinicians should approach anxiety assessment and when advanced machine learning methods may add value in mental health care. Consistent with existing diagnostic guidelines [4], our cross-sectional results indicate that readily available psychosocial assessments, such as self-reported symptoms of anxiety, depression, stress, and childhood adversity, provide a strong classification signal for clinically relevant anxiety phenotypes. Following a stepwise translational model, machine-learning integrating imaging features and other biological measures could be considered after initial symptom assessment to inform decision points not fully resolved by standard evaluation, such as individualized treatment planning, specifically when patients show ambiguous treatment response, relapse risk estimation, or the management of complex comorbidity [92]. As emphasized in translational frameworks for psychiatric machine learning, clinical utility arises when models inform prognosis, treatment selection, or risk stratification, rather than merely reproducing existing diagnostic categories [93]. When validated in longitudinal and clinically characterized samples, such models and their derived subgroup patterns could be systematically considered in the development and refinement of therapeutic guidelines, particularly in the context of stratified and stepped-care approaches [94, 95]. Thus, machine learning should be viewed not as a replacement for clinical judgment, but as a complementary tool to enhance precision in pre-disorder or later diagnostic and therapeutic stages.

Finally, we would like to point out some limitations and potential directions for future research. First, although the sample size was significantly larger than many previous studies, the population was still limited to an epidemiological cohort consisting of residents of Germany, which may affect the generalizability of the results to other geographical populations and to clinical populations with diagnosed anxiety disorders displaying higher symptom severity and potentially higher degrees of neuroanatomical changes. Consequently, we might underestimate the contribution of structural imaging features to GAD and panic attacks classification, and a higher grade of anxiety symptomatology might improve the overall performance of neuroanatomical variables in complex models. Moreover, future studies should aim to replicate these findings in more ethnically, clinically and geographically diverse samples to better understand cross-ethnic differences in the neurobiological underpinnings of GAD and panic attacks. Furthermore, the low prevalence of GAD and panic attacks may have limited the predictive validity in subgroup analyses.

Second, the study was based on cross-sectional data, which limits the ability to draw causal conclusions and to make temporal predictions. Longitudinal studies that track brain structure and symptom development over time are needed to assess how structural changes in the brain might influence or be influenced by the progression of anxiety disorders. Exploring these additional factors in future research could lead to a more comprehensive understanding of the neurological correlates of GAD and panic-related pathology. Along these lines, while the present work focuses on symptom classification, the nomothetic networks psychiatry perspective highlights the value of future models that also test pathway-like relations among psychosocial risk factors, brain measures, and symptom dimensions. In addition, future studies could extend the current multimodal feature set by incorporating peripheral biological markers (e.g., oxidative/nitrosative stress, lipid peroxidation, antioxidant defenses, inflammatory or endocrine markers), which have been implicated in anxiety-related phenotypes and may provide complementary information beyond neuroimaging and questionnaire-based measures [96].

Third, despite the use of machine learning algorithms, the predictive power of neuroimaging data alone was relatively low compared to psychosocial variables. This suggests that structural MRI using the imaging protocol, gray/white matter contrast and spatial resolution used in the NAKO study may not fully capture the complexity of anxiety disorders. Functional neuroimaging or multimodal approaches, including genetics, may provide additional layers of information that could improve predictive accuracy.

Along these lines, we must address the fact that some of the most important features identified by the machine learning analyses did not show significant relationships with the outcome variables when examined using a conventional logistic regression analysis. Rather than indicating contradictory results, this reflects differences in analytical goals and assumptions between univariate or low-dimensional association-based approaches and multivariate classification models. While this makes interpretation of the results somewhat more challenging, it also highlights the advantages of machine learning approaches in detecting associations that would be difficult to uncover through conventional methods. The applied machine learning approaches can accommodate numerous multicollinear predictors, a known limitation of traditional analyses. Accordingly, we view machine learning as a complementary methodological approach that is particularly well suited for high-dimensional data, rather than as a replacement for conventional statistical analyses [97].

### Conclusion

We applied machine learning techniques to a large cohort dataset to classify the presence vs. absence of GAD and panic attacks. The results indicate that psychosocial variables, including symptoms of depression, childhood trauma, and psychological distress, are more predictive of GAD and panic attacks than neuroimaging measures of gray matter volume. While the integration of neuroimaging data improved specificity for GAD and panic attacks, it did not significantly enhance overall predictive performance. These findings underscore the importance of considering psychological and demographic factors when developing predictive models for anxiety disorders, while also highlighting the limited standalone predictive utility of state-of-the-art structural MRI data. At the same time, neuroimaging features provided complementary, conditionally informative signals within multivariate models. We therefore advocate multimodal approaches and longitudinal designs to better understand the dynamic interplay between neurobiological factors and clinical symptoms, ultimately advancing the identification of objective and measurable biomarkers for anxiety disorders.

## Supplementary information

Supplementary Material Legends

S1 Correlational Analyses between pre-selected neural variables and GAD symptoms (S1a) and panic attacks (S1b).

S2 GAD variable Importance - Psychosocial model (P)

S3 Panic attacks variable importance - Psychosocial model (P)

S4 GAD variable importance - Neuroimaging (N) model

S5 Panic attacks variable importance - Neuroimaging (N) model

S6 GAD variable importance - Combined (P+N) model

S7 Panic attacks variable importance - Combined (P+N) model

S8 Model hyperparameters

## Data Availability

The data that support the findings of this study are part of the German National Cohort (NAKO Gesundheitsstudie). NAKO data are subject to the EU / EEA General Data Protection Regulation and to the NAKO Terms of Use. They can therefore not be deposited in a public repository. Qualified researchers affiliated to EU or EEA institutions may apply for access through the NAKO TransferHub (https://transfer.nako.de). Applications are evaluated by the NAKO Use & Access Committee; successful applicants must sign a Data-Use Agreement and cover associated handling fees.

## Footnotes

1 In this context, “predict” does not imply a temporal or causal forecast. Instead, it follows the common usage in machine learning and statistics, referring to the explanatory value of a model. The term “predict” is used throughout this paper in that sense, unless explicitly indicated otherwise.

2 This combined criterion was used to increase specificity and reduce over-classification of isolated panic attacks, given that only the screening component of the PHQ-Panic scale was available and a full diagnostic assessment could not be performed.

## Code availability

Analysis code is available at https://osf.io/wt9yf/.
