# Supplementary material for: Multimodal phenotypic classification of generalized anxiety and panic using structural MRI data and psychosocial factors: machine learning results from the German National Cohort (NAKO) study
Source: Transl Psychiatry. 2026 May 28;16:287. doi: 10.1038/s41398-026-04131-1 (PMC13219414; doi:10.1038/s41398-026-04131-1)
Supplement: Supplementary file 1 — Supplementary Material Legends [file 41398_2026_4131_MOESM1_ESM.docx]

S1. Correlational Analyses between pre-selecte neural variables and GAD symptoms (S1a) and panic attacks (S1b).

S2. GAD variable Importance - Psychosocial model (P).

S3. Panic attacks variable importance - Psychosocial model (P).

S4. GAD variable importance - Neuroimaging (N) model.

S5. Panic attacks variable importance - Neuroimaging (N) model.

S6. GAD variable importance - Combined (P+N) model.

S7. Panic attacks variable importance - Combined (P+N) model.

S8. Model hyperparameters.
