# Supplementary material for: Multimodal phenotypic classification of generalized anxiety and panic using structural MRI data and psychosocial factors: machine learning results from the German National Cohort (NAKO) study
Source: Transl Psychiatry. 2026 May 28;16:287. doi: 10.1038/s41398-026-04131-1 (PMC13219414; doi:10.1038/s41398-026-04131-1)
Supplement: Supplementary file 2 — S1 Correlational Analyses between pre-selected neural variables and GAD symptoms (S1a) and panic attacks (S1b). [file 41398_2026_4131_MOESM2_ESM.docx]

**S1. Correlational analyses of preselected neuroimaging variables and GAD symptoms and panic attacks**

As supplementary, hypothesis-oriented analyses, we examined univariate associations between a theory-driven, preselected set of 93 neuroimaging variables and clinically relevant GAD symptoms and panic attacks using Pearson correlations. The selected neuroimaging variables comprised global brain metrics (e.g., total gray and white matter volumes), regional gray matter volumes (e.g., insula, amygdala, hippocampus, and cingulate cortex), surface areas, subcortical structures, and mean cortical thickness measures in brain regions implicated in anxiety-related processes (Craske et al., 2017; Harrewijn et al., 2021; Pessoa, 2023). Additionally, we examined network-specific gray matter volumes, such as those associated with the Default Mode and Salience Networks, as well as limbic regions implicated in emotional regulation (Alves et al., 2019; Catani et al., 2013).

To examine associations between neuroimaging variables and anxiety phenotypes beyond variance shared with age and overlapping psychopathology, we applied a sequential covariate-adjustment approach (Snoek et al., 2019). For the correlations with GAD symptoms, the analyses were controlled for age, depressive symptoms (PHQ-9 score), panic attacks, childhood trauma, and scanner site. For the correlations with panic attacks, the analyses were controlled for age, depressive symptoms (PHQ-9 score), clinically relevant GAD symptoms (GAD-7 ≥ 10), childhood trauma, and scanner type. All correlation analyses were stratified by sex, given established sex differences in the prevalence and neurobiological correlates of anxiety-related phenotypes (Jalnapurkar et al., 2018), all correlation analyses were stratified by sex. To control for multiple comparisons, p-values were adjusted using the false discovery rate (FDR) procedure according to Benjamini and Hochberg (1995), with a nominal FDR of 5% applied separately within the female and male groups. The results of these analyses are depicted in Tables S1 (clinically relevant GAD symptoms) and S2 (panic attacks).

**Table S1.** Correlations between clinically relevant GAD symptoms (GAD-7 ≥10) and various neuroimaging variables, corrected for relevant confounders, stratified by sex.

| Variable | female | | male | |
| --- | --- | --- | --- | --- |
|  | ***r*** | ***p*_FDR_** | ***r*** | ***p*_FDR_** |
| Gray matter volume of left Default Mode network, A, dorsal prefrontal cortex, region 1 | 0.016 | **0.001*** | 0.000 | 0.391 |
| Gray matter volume of left Default Mode network, A, medial prefrontal cortex, region 1 | 0.023 | **0.000*** | 0.012 | 0.481 |
| Gray matter volume of left Default Mode network, A, precuneus posterior cingulate cortex, region 1 | 0.010 | **0.011*** | 0.029 | **0.005*** |
| Gray matter volume of left Default Mode network, B, dorsal prefrontal cortex, region 1 | 0.028 | **0.000*** | 0.005 | 0.099 |
| Gray matter volume of left Default Mode network, B, inferior parietal lobule, region 1 | 0.017 | **0.000*** | 0.014 | **0.008*** |
| Gray matter volume of left Default Mode network, B, lateral prefrontal cortex, region 1 | 0.019 | **0.006*** | 0.017 | **0.004*** |
| Gray matter volume of left Default Mode network, B, ventral prefrontal cortex, region 1 | 0.010 | 0.096 | 0.011 | 0.481 |
| Gray matter volume of left Default Mode network, B, ventral prefrontal cortex, region 2 | 0.015 | **0.016*** | 0.019 | **0.008*** |
| Gray matter volume of left Default Mode network, C, parahippocampal cortex, region 1 | 0.021 | 0.701 | 0.013 | 0.481 |
| Gray matter volume of left Limbic network, B, orbital frontal cortex, region 1 | 0.013 | 0.473 | 0.023 | **0.008*** |
| Gray matter volume of left Salience Ventral Attention network, A, insula, region 2 | 0.021 | 0.097 | 0.012 | 0.132 |
| Gray matter volume of left Salience Ventral Attention network, A, parietal medial, region 1 | 0.029 | **0.019*** | 0.010 | 0.489 |
| Gray matter volume of left Salience Ventral Attention network, A, parietal operculum, region 1 | 0.027 | **0.002*** | 0.027 | **0.001*** |
| Gray matter volume of left Salience Ventral Attention network, B, lateral prefrontal cortex, region 1 | 0.018 | **0.001*** | 0.001 | 0.786 |
| Gray matter volume of left Salience Ventral Attention network, B, medial posterior prefrontal cortex, region 1 | 0.028 | **0.003*** | 0.006 | 0.786 |
| Gray matter volume of right Default Mode network, A, dorsal prefrontal cortex, region 1 | 0.020 | **0.001*** | 0.007 | 0.155 |
| Gray matter volume of right Default Mode network, A, inferior parietal lobule, region 1 | 0.016 | **0.011*** | 0.035 | **0.000*** |
| Gray matter volume of right Default Mode network, A, medial prefrontal cortex, region 1 | 0.034 | **0.001*** | 0.006 | 0.249 |
| Gray matter volume of right Default Mode network, A, precuneus posterior cingulate cortex, region 1 | 0.016 | **0.001*** | 0.021 | **0.014*** |
| Gray matter volume of right Default Mode network, B, dorsal prefrontal cortex, region 1 | 0.024 | **0.015*** | 0.015 | 0.083 |
| Gray matter volume of right Default Mode network, B, ventral prefrontal cortex, region 1 | 0.019 | **0.002*** | 0.007 | 0.264 |
| Gray matter volume of right Default Mode network, B, ventral prefrontal cortex, region 2 | 0.028 | **0.000*** | 0.019 | **0.034*** |
| Gray matter volume of right Default Mode network, C, parahippocampal cortex, region 1 | 0.017 | **0.008*** | 0.025 | **0.001*** |
| Gray matter volume of right Default Mode network, C, retrosplenia cortexl, region 1 | 0.018 | 0.129 | 0.021 | 0.053 |
| Gray matter volume of right Limbic network, A, temporal pole, region 1 | 0.031 | **0.000*** | 0.021 | 0.086 |
| Gray matter volume of right Limbic network, B, orbital frontal cortex, region 1 | 0.014 | **0.014*** | 0.018 | **0.003*** |
| Gray matter volume of right Salience Ventral Attention network, A, frontal medial, region 1 | 0.015 | 0.373 | 0.015 | 0.242 |
| Gray matter volume of right Salience Ventral Attention network, A, insula, region 1 | 0.020 | **0.011*** | 0.020 | 0.095 |
| Gray matter volume of right Salience Ventral Attention network, A, parietal medial, region 1 | 0.019 | **0.030*** | 0.011 | **0.017*** |
| Gray matter volume of right Salience Ventral Attention network, A, parietal operculum, region 1 | 0.012 | **0.001*** | 0.020 | 0.112 |
| Gray matter volume of right Salience Ventral Attention network, B, inferior parietal lobule, region 1 | 0.015 | **0.035*** | 0.024 | **0.002*** |
| Gray matter volume of right Salience Ventral Attention network, B, lateral prefrontal cortex, region 1 | 0.022 | **0.006*** | 0.003 | 0.837 |
| Gray matter volume of right Salience Ventral Attention network, B, medial posterior prefrontal cortex, region 1 | 0.014 | **0.035*** | 0.017 | 0.269 |
| Gray matter volume of the left caudal anterior cingulate cortex | 0.012 | **0.030*** | 0.015 | 0.051 |
| Gray matter volume of the left insula | 0.061 | **0.000*** | 0.053 | **0.000*** |
| Gray matter volume of the left isthmus cingulate cortex | 0.051 | **0.000*** | 0.029 | **0.001*** |
| Gray matter volume of the left medial orbitofrontal cortex | 0.055 | **0.000*** | 0.057 | **0.000*** |
| Gray matter volume of the left parahippocampal gyrus | 0.027 | **0.000*** | 0.027 | **0.003*** |
| Gray matter volume of the left rostral anterior cingulate cortex | 0.055 | **0.000*** | 0.052 | **0.000*** |
| Gray matter volume of the leftposterior cingulate cortex | 0.046 | **0.000*** | 0.032 | **0.000*** |
| Gray matter volume of the right caudal anterior cingulate cortex | 0.024 | **0.019*** | 0.025 | **0.000*** |
| Gray matter volume of the right insula | 0.059 | **0.000*** | 0.061 | **0.000*** |
| Gray matter volume of the right isthmus cingulate cortex | 0.047 | **0.000*** | 0.041 | **0.000*** |
| Gray matter volume of the right parahippocampal gyrus | 0.030 | **0.000*** | 0.026 | 0.155 |
| Gray matter volume of the right posterior cingulate cortex | 0.045 | **0.000*** | 0.038 | **0.000*** |
| Gray matter volume of the right rostral anterior cingulate cortex | 0.043 | **0.000*** | 0.027 | **0.001*** |
| Left hemispheric cerebral white matter volume | 0.088 | **0.000*** | 0.061 | **0.000*** |
| Mean cortical thickness of the left caudal anterior cingulate cortex | 0.000 | 0.701 | -0.005 | 0.914 |
| Mean cortical thickness of the left caudal middle frontal gyrus | 0.007 | 0.983 | 0.001 | 0.615 |
| Mean cortical thickness of the left insula | -0.005 | 0.743 | 0.009 | **0.021*** |
| Mean cortical thickness of the left isthmus cingulate cortex | -0.003 | 0.148 | -0.001 | 0.814 |
| Mean cortical thickness of the left parahippocampal gyrus | -0.003 | 0.969 | -0.003 | 0.217 |
| Mean cortical thickness of the left rostral anterior cingulate cortex | -0.005 | 0.856 | -0.005 | 0.561 |
| Mean cortical thickness of the leftposterior cingulate cortex | -0.001 | 0.975 | 0.001 | 0.647 |
| Mean cortical thickness of the right caudal anterior cingulate cortex | -0.008 | 0.351 | -0.017 | 0.259 |
| Mean cortical thickness of the right hemisphere | -0.002 | 0.901 | 0.002 | 0.750 |
| Mean cortical thickness of the right insula | 0.002 | 0.848 | -0.006 | 0.561 |
| Mean cortical thickness of the right isthmus cingulate cortex | -0.009 | 0.421 | -0.013 | 0.554 |
| Mean cortical thickness of the right medial orbitofrontal cortex | 0.005 | 0.969 | 0.007 | 0.951 |
| Mean cortical thickness of the right parahippocampal gyrus | -0.009 | 0.563 | -0.005 | 0.354 |
| Mean cortical thickness of the right posterior cingulate cortex | -0.006 | 0.238 | -0.008 | 0.482 |
| Mean cortical thickness of the right rostral anterior cingulate cortex | 0.005 | 0.664 | 0.004 | 0.953 |
| Subcortical gray matter volume | 0.086 | **0.000*** | 0.072 | **0.000*** |
| Surface area of the left caudal anterior cingulate cortex | 0.026 | **0.019*** | 0.033 | **0.000*** |
| Surface area of the left caudal middle frontal gyrus | 0.034 | **0.000*** | 0.029 | **0.003*** |
| Surface area of the left hemisphere | 0.082 | **0.000*** | 0.073 | **0.000*** |
| Surface area of the left insula | 0.048 | **0.000*** | 0.055 | **0.000*** |
| Surface area of the left isthmus cingulate cortex | 0.049 | **0.000*** | 0.051 | **0.000*** |
| Surface area of the left parahippocampal gyrus | 0.043 | **0.000*** | 0.041 | **0.013*** |
| Surface area of the left rostral anterior cingulate cortex | 0.043 | **0.000*** | 0.029 | **0.000*** |
| Surface area of the leftposterior cingulate cortex | 0.037 | **0.000*** | 0.044 | **0.000*** |
| Surface area of the right caudal anterior cingulate cortex | 0.026 | **0.020*** | 0.034 | **0.000*** |
| Surface area of the right hemisphere | 0.082 | **0.000*** | 0.073 | **0.000*** |
| Surface area of the right insula | 0.048 | **0.000*** | 0.055 | **0.000*** |
| Surface area of the right isthmus cingulate cortex | 0.048 | **0.000*** | 0.051 | **0.000*** |
| Surface area of the right posterior cingulate cortex | 0.036 | **0.000*** | 0.044 | **0.000*** |
| Surface area of the right rostral anterior cingulate cortex | 0.043 | **0.000*** | 0.029 | **0.000*** |
| Total cerebral white matter volume | 0.088 | **0.000*** | 0.062 | **0.000*** |
| Total gray matter volume | 0.098 | **0.000*** | 0.076 | **0.000*** |
| Total volume of white matter lesions | -0.008 | **0.030*** | -0.019 | **0.001*** |
| Volume of the Brain stem | 0.087 | **0.000*** | 0.056 | **0.000*** |
| Volume of the left Accumbens area | 0.034 | **0.001*** | 0.041 | **0.005*** |
| Volume of the left Amygdala | 0.063 | **0.000*** | 0.056 | **0.000*** |
| Volume of the left Hippocampus | 0.066 | **0.000*** | 0.053 | **0.000*** |
| Volume of the left Thalamus | 0.070 | **0.000*** | 0.052 | **0.000*** |
| Volume of the left ventral diencephalon | 0.087 | **0.000*** | 0.064 | **0.000*** |
| Volume of the right Accumbens area | 0.035 | **0.000*** | 0.037 | **0.000*** |
| Volume of the right Amygdala | 0.075 | **0.000*** | 0.051 | **0.000*** |
| Volume of the right Hippocampus | 0.060 | **0.000*** | 0.051 | **0.000*** |
| Volume of the right Thalamus | 0.071 | **0.000*** | 0.056 | **0.000*** |
| Volume of the right ventral diencephalon | 0.087 | **0.000*** | 0.063 | **0.000*** |
| left cortical gray matter volume | 0.086 | **0.000*** | 0.066 | **0.000*** |
| right cortical gray matter volume | 0.087 | **0.000*** | 0.066 | **0.000*** |

*Note. Correlations are corrected for age, depressive symptoms (PHQ-9 score), panic attacks, childhood trauma, and scanner site; p-values were adjusted following the FDR procedure (Benjamini & Hochberg, 1995), with a nominal FDR of 5% applied separately for males and females.*

**Table S2.** Correlations between panic attacks and various neuroimaging variables, corrected for relevant confounders, stratified by sex.

| Variable | female | | male | |
| --- | --- | --- | --- | --- |
|  | ***r*** | ***p*_FDR_** | ***r*** | ***p*_FDR_** |
| Gray matter volume of left Default Mode network, A, dorsal prefrontal cortex, region 1 | 0.013 | **0.040*** | 0.004 | 0.298 |
| Gray matter volume of left Default Mode network, A, medial prefrontal cortex, region 1 | 0.003 | 0.145 | -0.009 | 0.912 |
| Gray matter volume of left Default Mode network, A, precuneus posterior cingulate cortex, region 1 | 0.018 | **0.007*** | 0.009 | 0.108 |
| Gray matter volume of left Default Mode network, B, dorsal prefrontal cortex, region 1 | 0.009 | **0.040*** | 0.000 | 0.371 |
| Gray matter volume of left Default Mode network, B, inferior parietal lobule, region 1 | 0.027 | **0.000*** | 0.007 | 0.091 |
| Gray matter volume of left Default Mode network, B, lateral prefrontal cortex, region 1 | -0.017 | 0.617 | 0.019 | **0.005*** |
| Gray matter volume of left Default Mode network, B, ventral prefrontal cortex, region 1 | 0.013 | 0.097 | 0.007 | 0.354 |
| Gray matter volume of left Default Mode network, B, ventral prefrontal cortex, region 2 | 0.007 | 0.108 | 0.009 | 0.091 |
| Gray matter volume of left Default Mode network, C, parahippocampal cortex, region 1 | 0.011 | 0.147 | -0.001 | 0.844 |
| Gray matter volume of left Limbic network, B, orbital frontal cortex, region 1 | 0.017 | **0.040*** | -0.002 | 0.951 |
| Gray matter volume of left Salience Ventral Attention network, A, insula, region 2 | 0.011 | 0.076 | -0.010 | 0.611 |
| Gray matter volume of left Salience Ventral Attention network, A, parietal medial, region 1 | -0.001 | 0.547 | -0.003 | 0.825 |
| Gray matter volume of left Salience Ventral Attention network, A, parietal operculum, region 1 | 0.004 | 0.085 | 0.009 | 0.061 |
| Gray matter volume of left Salience Ventral Attention network, B, lateral prefrontal cortex, region 1 | 0.019 | **0.011*** | 0.000 | 0.607 |
| Gray matter volume of left Salience Ventral Attention network, B, medial posterior prefrontal cortex, region 1 | 0.016 | **0.023*** | -0.006 | 0.921 |
| Gray matter volume of right Default Mode network, A, dorsal prefrontal cortex, region 1 | 0.018 | **0.007*** | 0.002 | 0.383 |
| Gray matter volume of right Default Mode network, A, inferior parietal lobule, region 1 | 0.003 | 0.147 | 0.012 | **0.036*** |
| Gray matter volume of right Default Mode network, A, medial prefrontal cortex, region 1 | 0.003 | 0.158 | 0.002 | 0.347 |
| Gray matter volume of right Default Mode network, A, precuneus posterior cingulate cortex, region 1 | 0.001 | 0.272 | 0.007 | 0.135 |
| Gray matter volume of right Default Mode network, B, dorsal prefrontal cortex, region 1 | -0.016 | 0.530 | 0.008 | 0.148 |
| Gray matter volume of right Default Mode network, B, ventral prefrontal cortex, region 1 | 0.011 | **0.046*** | 0.000 | 0.531 |
| Gray matter volume of right Default Mode network, B, ventral prefrontal cortex, region 2 | 0.006 | 0.080 | 0.000 | 0.383 |
| Gray matter volume of right Default Mode network, C, parahippocampal cortex, region 1 | -0.003 | 0.406 | 0.015 | **0.012*** |
| Gray matter volume of right Default Mode network, C, retrosplenia cortexl, region 1 | -0.010 | 0.910 | 0.013 | 0.060 |
| Gray matter volume of right Limbic network, A, temporal pole, region 1 | 0.016 | **0.010*** | 0.008 | 0.108 |
| Gray matter volume of right Limbic network, B, orbital frontal cortex, region 1 | -0.002 | 0.337 | -0.001 | 0.512 |
| Gray matter volume of right Salience Ventral Attention network, A, frontal medial, region 1 | 0.003 | 0.484 | 0.008 | 0.273 |
| Gray matter volume of right Salience Ventral Attention network, A, insula, region 1 | 0.005 | 0.161 | 0.000 | 0.595 |
| Gray matter volume of right Salience Ventral Attention network, A, parietal medial, region 1 | 0.006 | 0.145 | 0.009 | 0.131 |
| Gray matter volume of right Salience Ventral Attention network, A, parietal operculum, region 1 | -0.001 | 0.477 | 0.009 | 0.131 |
| Gray matter volume of right Salience Ventral Attention network, B, inferior parietal lobule, region 1 | 0.006 | 0.094 | 0.018 | **0.006*** |
| Gray matter volume of right Salience Ventral Attention network, B, lateral prefrontal cortex, region 1 | 0.006 | 0.178 | 0.000 | 0.607 |
| Gray matter volume of right Salience Ventral Attention network, B, medial posterior prefrontal cortex, region 1 | 0.012 | 0.052 | 0.007 | 0.219 |
| Gray matter volume of the left caudal anterior cingulate cortex | 0.005 | 0.202 | 0.011 | 0.091 |
| Gray matter volume of the left insula | 0.011 | **0.000*** | 0.018 | **0.000*** |
| Gray matter volume of the left isthmus cingulate cortex | 0.016 | **0.001*** | 0.016 | **0.003*** |
| Gray matter volume of the left medial orbitofrontal cortex | 0.011 | **0.000*** | 0.020 | **0.000*** |
| Gray matter volume of the left parahippocampal gyrus | 0.007 | 0.052 | 0.008 | 0.082 |
| Gray matter volume of the left rostral anterior cingulate cortex | 0.022 | **0.000*** | 0.015 | **0.002*** |
| Gray matter volume of the leftposterior cingulate cortex | 0.021 | **0.000*** | 0.008 | **0.016*** |
| Gray matter volume of the right caudal anterior cingulate cortex | 0.014 | **0.023*** | -0.005 | 0.879 |
| Gray matter volume of the right insula | 0.008 | **0.000*** | 0.016 | **0.000*** |
| Gray matter volume of the right isthmus cingulate cortex | 0.028 | **0.000*** | 0.010 | **0.021*** |
| Gray matter volume of the right parahippocampal gyrus | 0.016 | **0.008*** | 0.002 | 0.314 |
| Gray matter volume of the right posterior cingulate cortex | 0.021 | **0.000*** | 0.015 | **0.001*** |
| Gray matter volume of the right rostral anterior cingulate cortex | 0.008 | **0.036*** | 0.005 | 0.124 |
| Left hemispheric cerebral white matter volume | 0.024 | **0.000*** | 0.010 | **0.000*** |
| Mean cortical thickness of the left caudal anterior cingulate cortex | -0.002 | 0.843 | 0.005 | 0.698 |
| Mean cortical thickness of the left caudal middle frontal gyrus | 0.003 | 0.758 | -0.006 | 0.574 |
| Mean cortical thickness of the left insula | -0.008 | 0.758 | -0.006 | 0.814 |
| Mean cortical thickness of the left isthmus cingulate cortex | -0.004 | 0.617 | 0.012 | 0.298 |
| Mean cortical thickness of the left parahippocampal gyrus | 0.001 | 0.741 | -0.001 | 0.940 |
| Mean cortical thickness of the left rostral anterior cingulate cortex | 0.003 | 0.910 | -0.005 | 0.515 |
| Mean cortical thickness of the leftposterior cingulate cortex | -0.014 | 0.303 | 0.001 | 0.772 |
| Mean cortical thickness of the right caudal anterior cingulate cortex | -0.005 | 0.484 | -0.014 | 0.091 |
| Mean cortical thickness of the right hemisphere | 0.001 | 0.842 | 0.002 | 0.814 |
| Mean cortical thickness of the right insula | 0.000 | 0.842 | -0.013 | 0.263 |
| Mean cortical thickness of the right isthmus cingulate cortex | 0.003 | 0.842 | 0.007 | 0.599 |
| Mean cortical thickness of the right medial orbitofrontal cortex | 0.001 | 0.969 | -0.010 | 0.273 |
| Mean cortical thickness of the right parahippocampal gyrus | -0.012 | 0.402 | -0.003 | 0.912 |
| Mean cortical thickness of the right posterior cingulate cortex | -0.011 | 0.206 | 0.000 | 0.818 |
| Mean cortical thickness of the right rostral anterior cingulate cortex | -0.004 | 0.731 | -0.009 | 0.347 |
| Subcortical gray matter volume | 0.029 | **0.000*** | 0.012 | **0.001*** |
| Surface area of the left caudal anterior cingulate cortex | 0.010 | **0.046*** | -0.002 | 0.607 |
| Surface area of the left caudal middle frontal gyrus | -0.012 | 0.741 | 0.003 | 0.160 |
| Surface area of the left hemisphere | 0.020 | **0.000*** | 0.024 | **0.000*** |
| Surface area of the left insula | 0.004 | **0.001*** | 0.012 | **0.000*** |
| Surface area of the left isthmus cingulate cortex | 0.023 | **0.000*** | 0.012 | **0.006*** |
| Surface area of the left parahippocampal gyrus | 0.023 | **0.000*** | 0.014 | **0.010*** |
| Surface area of the left rostral anterior cingulate cortex | 0.009 | **0.023*** | 0.007 | 0.060 |
| Surface area of the leftposterior cingulate cortex | 0.013 | **0.002*** | 0.018 | **0.001*** |
| Surface area of the right caudal anterior cingulate cortex | 0.010 | **0.047*** | -0.002 | 0.607 |
| Surface area of the right hemisphere | 0.020 | **0.000*** | 0.024 | **0.000*** |
| Surface area of the right insula | 0.004 | **0.001*** | 0.012 | **0.000*** |
| Surface area of the right isthmus cingulate cortex | 0.023 | **0.000*** | 0.012 | **0.006*** |
| Surface area of the right posterior cingulate cortex | 0.013 | **0.002*** | 0.018 | **0.001*** |
| Surface area of the right rostral anterior cingulate cortex | 0.008 | **0.024*** | 0.007 | 0.060 |
| Total cerebral white matter volume | 0.025 | **0.000*** | 0.011 | **0.000*** |
| Total gray matter volume | 0.037 | **0.000*** | 0.020 | **0.000*** |
| Total volume of white matter lesions | -0.007 | 0.178 | -0.011 | 0.060 |
| Volume of the Brain stem | 0.032 | **0.000*** | 0.009 | **0.001*** |
| Volume of the left Accubmens area | 0.011 | **0.046*** | 0.012 | 0.073 |
| Volume of the left Amygdala | 0.020 | **0.000*** | 0.009 | **0.007*** |
| Volume of the left Hippocampus | 0.015 | **0.000*** | -0.001 | 0.076 |
| Volume of the left Thalamus | 0.030 | **0.000*** | -0.002 | 0.248 |
| Volume of the left ventral diencephalon | 0.033 | **0.000*** | 0.010 | **0.001*** |
| Volume of the right Accumbens area | 0.003 | 0.102 | 0.013 | **0.029*** |
| Volume of the right Amygdala | 0.021 | **0.000*** | 0.011 | **0.001*** |
| Volume of the right Hippocampus | 0.013 | **0.000*** | 0.006 | **0.013*** |
| Volume of the right Thalamus | 0.030 | **0.000*** | -0.002 | 0.151 |
| Volume of the right ventral diencephalon | 0.038 | **0.000*** | 0.012 | **0.001*** |
| left cortical gray matter volume | 0.028 | **0.000*** | 0.016 | **0.000*** |
| right cortical gray matter volume | 0.031 | **0.000*** | 0.018 | **0.000*** |

*Note. Correlations are corrected for age, depressive symptoms (PHQ-9 score), clinically relevant GAD symptoms (*GAD-7 ≥ 10*), childhood trauma, and scanner site; p-values were adjusted following the FDR procedure (Benjamini & Hochberg, 1995), with a nominal FDR of 5% applied separately for males and females.*

References

Alves, P. N., Foulon, C., Karolis, V., Bzdok, D., Margulies, D. S., Volle, E., & Thiebaut de Schotten, M. (2019). An improved neuroanatomical model of the default-mode network reconciles previous neuroimaging and neuropathological findings. *Communications Biology*, *2*(1), 1–14. https://doi.org/10.1038/s42003-019-0611-3

Benjamini, Y., & Hochberg, Y. (1995). Controlling the False Discovery Rate: A Practical and Powerful Approach to Multiple Testing. *Journal of the Royal Statistical Society. Series B (Methodological)*, *57*(1), 289–300.

Catani, M., Dell’Acqua, F., & Thiebaut de Schotten, M. (2013). A revised limbic system model for memory, emotion and behaviour. *Neuroscience & Biobehavioral Reviews*, *37*(8), 1724–1737. https://doi.org/10.1016/j.neubiorev.2013.07.001

Craske, M. G., Stein, M. B., Eley, T. C., Milad, M. R., Holmes, A., Rapee, R. M., & Wittchen, H.-U. (2017). Anxiety disorders. *Nature Reviews Disease Primers*, *3*(1), 1–19. https://doi.org/10.1038/nrdp.2017.24

Harrewijn, A., Cardinale, E. M., Groenewold, N. A., Bas-Hoogendam, J. M., Aghajani, M., Hilbert, K., Cardoner, N., Porta-Casteràs, D., Gosnell, S., Salas, R., Jackowski, A. P., Pan, P. M., Salum, G. A., Blair, K. S., Blair, J. R., Hammoud, M. Z., Milad, M. R., Burkhouse, K. L., Phan, K. L., … Pine, D. S. (2021). Cortical and subcortical brain structure in generalized anxiety disorder: Findings from 28 research sites in the ENIGMA-Anxiety Working Group. *Translational Psychiatry*, *11*(1), 1–15. https://doi.org/10.1038/s41398-021-01622-1

Jalnapurkar, I., Allen, M., & Pigott, T. (2018). Sex differences in anxiety disorders: A review. *J Psychiatry Depress Anxiety*, *4*(12), 3–16.

Pessoa, L. (2023). How many brain regions are needed to elucidate the neural bases of fear and anxiety? *Neuroscience & Biobehavioral Reviews*, *146*, 105039. https://doi.org/10.1016/j.neubiorev.2023.105039

Snoek, L., Miletić, S., & Scholte, H. S. (2019). How to control for confounds in decoding analyses of neuroimaging data. *NeuroImage*, *184*, 741–760. https://doi.org/10.1016/j.neuroimage.2018.09.074
